# Comparative experimental evaluation and empirical modeling of advanced oxidation processes for organic compounds removal from cosmetic wastewater with statistical assessment

**DOI:** 10.1038/s41598-025-18131-6

**Published:** 2025-09-29

**Authors:** Omnia Ismail, Taha Ebrahim Farrag, Y. Reda, Riham Hazzaa

**Affiliations:** 1 Chemical Engineering Department, Faculty of Engineering, Port Said University, Port Said, Egypt; 2 Chemical Engineering Department, Canal High Institute of Engineering and Technology, Suez, Egypt

**Keywords:** Cosmetic wastewater, Photo fenton, COD removal, Advanced oxidation, Biodegradability, Statistical modeling, Environmental sciences, Engineering

## Abstract

Cosmetic industry wastewater is characterized by high COD, recalcitrant organic compounds, and poor biodegradability, posing significant challenges to conventional biological treatment systems. This study addresses these challenges through a comprehensive evaluation of four advanced oxidation processes (AOPs): UV, UV/H₂O₂, Photo Fenton, and Photo Fenton like, aiming to identify a sustainable and scalable solution for treating real industrial wastewater collected from a cosmetics factory in Badr City, Egypt. Batch experiments were conducted under varied conditions of pH, hydrogen peroxide dosage, catalyst concentration (Fe^+2^ or Fe^+3^), and UV irradiation time. Among the tested processes, the Photo-Fenton system showed the highest performance, achieving 95.5% COD removal and enhancing the biodegradability index (BOD₅/COD) from 0.28 to 0.8 under optimized conditions (pH 3, 0.75 g/L Fe^+2^, 1 mL/L H₂O₂, 40 min). Kinetic modeling was applied to all tested AOPs, and the results indicated that pseudo-first-order kinetics best described the degradation behavior, confirming the role of hydroxyl radicals in organic removal. A multiple linear regression model (R² = 0.851) was also developed to predict COD removal efficiency based on process parameters and statistical analysis confirmed significance of the optimal conditions. Energy and cost evaluations identified the Photo-Fenton process as the most efficient and economically feasible option, with the lowest specific energy consumption and material costs per liter treated. These findings suggest that the Photo Fenton process is a viable pre-treatment option for cosmetics wastewater, particularly in decentralized or industrial treatment systems. Future work should focus on scaling the process and integrating it with biological treatment units to improve sustainability.

## Introduction

Wastewater generated by the cosmetics industry poses a significant environmental challenge due to its complex chemical composition and poor biodegradability^1,2^. It typically contains persistent organic pollutants (POPs), surfactants, synthetic fragrances, dyes, and preservatives, which are not effectively removed by conventional biological treatment systems^3,4^. As global production of personal care and cosmetic products continues to rise, there is growing pressure on industrial facilities to implement more sustainable and effective wastewater treatment solutions. In this context, advanced oxidation processes (AOPs) have attracted increasing attention due to their ability to generate hydroxyl radicals (•OH) capable of degrading a wide range of recalcitrant organics^5,6^.

Over the past 25 years, numerous studies have explored H₂O₂-based AOPs, including UV/H₂O₂, Fenton, and Photo-Fenton processes, for treating synthetic wastewater and specific pollutants^7,8^. For example, Shokri, A et al. (2018) used a UV/ZnO system to degrade Acid Red 283 dye in synthetic wastewater with over 99.5% removal at 60 mins^9^. Shokri, A. (2020) demonstrated the efficiency of UV/PDS activated by ferrous ions for toluene degradation from a typical synthetic wastewater^10^, while Bayat, A et al. (2020) optimized a Fe + 2/PMS system for the removal of p-Nitrotoluene in typical synthetic wastewater using full factorial design^11^. While these studies have advanced the field, most are limited to controlled laboratory conditions with idealized synthetic wastewater matrices. There remains a lack of practical investigations applying multiple AOP systems on real industrial wastewater.

To date, relatively few studies have examined the performance of AOPs using real, untreated wastewater from the cosmetics industry, despite its growing global footprint. Real wastewater differs significantly from synthetic models due to the presence of interfering substances, variable pH, and complex organic loads^12^. Therefore, a major gap exists in evaluating and optimizing AOPs under actual operating conditions using real industrial wastewater. Furthermore, most previous research has focused on a single process rather than conducting comparative evaluations under consistent conditions. Additionally, few studies integrate statistical modeling to quantify the influence of key operational variables or assess the economic feasibility of different treatment options. These limitations restrict the scalability and practical implementation of AOPs in industrial settings. This study addresses these gaps by:

1. Examining four AOPs: UV, UV/H₂O₂, Photo Fenton, and Photo Fenton like using real cosmetic wastewater collected from an Egyptian factory.
2. comparing each process in terms of COD removal, biodegradability enhancement, energy consumption, and material cost, providing a holistic assessment of their industrial viability.
3. Performing kinetic analysis to understand the reaction rate and mechanism of each process for optimizing COD removal.
4. Employing statistical modeling to identify optimal operational parameters (pH, H₂O₂ dosage, Fe^+2^/Fe^+3^ concentration, irradiation time) and evaluate their significance.

The findings contribute to bridging the gap between laboratory scale studies and practical applications of AOPs in real-world industrial environments. While this study is based in Egypt, its implications extend to developing countries and industrial zones worldwide that lack centralized treatment infrastructure. This research presents an integrated framework for selecting appropriate AOPs in the cosmetic and related industries. The study aims to support more sustainable industrial wastewater treatment strategies and inform future design and implementation of scalable AOP systems.

The innovation and contributions of this manuscript are:

- The study utilizes real, untreated wastewater from a functioning cosmetics factory, unlike many prior studies that used synthetic or diluted wastewater.
- It conducts a comparative experimental approach of four AOPs (UV, UV/H₂O₂, Photo Fenton, and Photo Fenton like) under identical conditions, which is rarely reported in literature.
- Empirical and statistical model are developed to assess the influence of key operational variables and predict COD removal performance. Unlike many previous works, this model enables quantitative prediction and optimization, not just descriptive analysis.
- The study includes cost and energy analysis, providing practical insights into process feasibility, beyond conventional lab-scale efficiency reporting.
- It addresses a neglected industrial sector (cosmetics) with growing environmental impact, offering broader relevance to developing countries and decentralized treatment systems.
- The integration of real industrial wastewater, empirical modeling, and cost/energy assessment makes the findings applicable on a broader, real-world industrial scale, especially in developing countries.

## Methodology

### Materials used

The use of chemicals included a variety of materials with distinct compositions and purities. The chemicals comprised hydrogen peroxide (30% concentration, density of 1.15 g/cm^3^) as an oxidizing agent and sulfuric acid (95–97% purity, density of 1.84 g/cm³) for adjusting pH. Ferrous sulphate heptahydrate (99% purity) and ferric chloride hexahydrate (99% purity) were utilized as a catalyst in the Photo Fenton and Photo Fenton like processes, respectively. Sodium hydroxide (48% purity) was applied to quench the reaction and neutralize residual oxidants before COD measurements.

Because different samples of the effluent were routinely analyzed and composed under close observation during operations and treatments, the manufacturing process generated a variety of wastewater type^13^. The intervals of collection were chosen to correspond with the variety of industrial activities carried out. The characteristics of cosmetics wastewater that was collected over time to create annual averages that were representative of the entire range of operations are shown in Table 1.

**Table 1:** Characteristics of used cosmetic Wastewater.

Analyzer Parameter	Average Value
Oils and fats (mg/L)	32
BOD_5_ (mg/L)	1960
COD (mg/L)	7000
Biodegradability (BOD_5_/COD)	0.28
pH	5.2-6

**Fig. 1:**
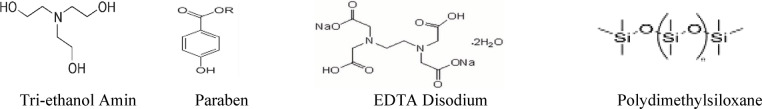
Chemical Formulas for Some Types of Organic Compounds Found in Cosmetic Wastewater.

Cosmetic wastewater which was selected in this research came from Egyptian factory in Bader City, Cairo, Egypt. This wastewater contains the following ingredients: stearic acid, cetyl alcohol, polydimethylsiloxane, glyceryl monostearate, dimethyl phthalate, diethyl phthalate, amphisol K, isopropyl myristate, Polysorbate 60, vitamin E acetate, EDTA disodium, propyl paraben, dimethicon 350, glycerin, triethanolamine, methyl paraben, Bi-pure, perfumes, D-panthenol, talc Powder, water and dyes such as iron oxide. Chemical formulas for some types of organic compounds found in cosmetic wastewater is shown in Fig. 1.

### Experimental setup

**Fig. 2:**
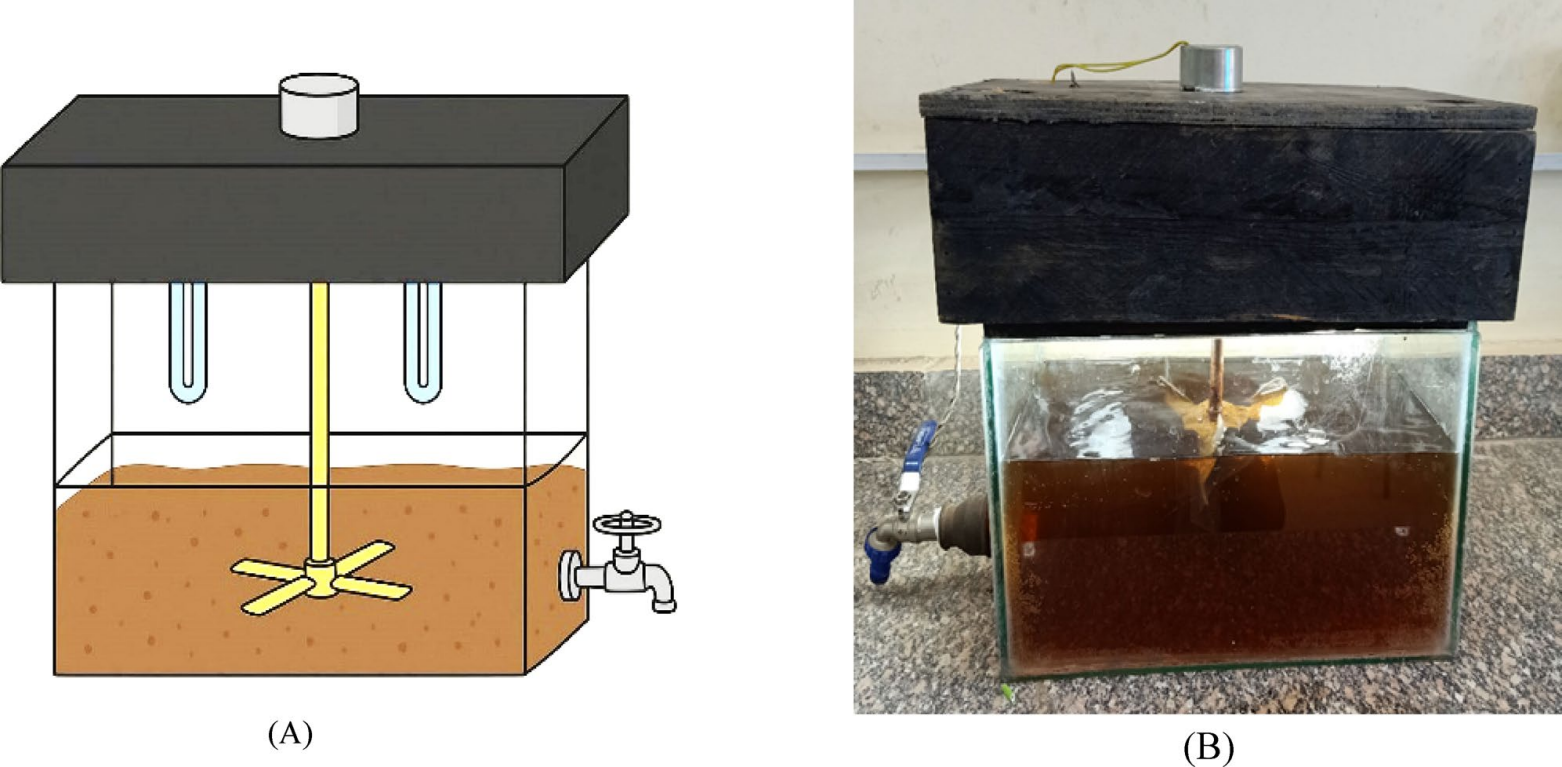
Schematic Diagram of Batch Reactor Used Photo Chemical Oxidation Processes (A) and Photographic View for The Actual Reactor (B).

The aim of the experimental work was to evaluate and compare the efficiency of (AOPs): UV photolysis, UV/H₂O₂, Photo Fenton, and Photo Fenton like in the treatment of real cosmetic wastewater. Each process was carried out in a batch reactor with a volume 1 L under varying conditions to determine the optimal operating parameters including pH, irradiation time, hydrogen peroxide dosage, and iron salt concentration. The reactor was made of quartz glass, that was available for the transfer of the radiation by two high pressure mercury lamps (TQ 75 W each), mounted symmetrically around the reactor to ensure uniform exposure. These lamps emit predominantly at 254 nm, which lies within the germicidal UV-C range, and are suitable for advanced oxidation reactions. The total UV power used was 150 W. Although the exact radiation flux was reported as approximately 2210 µW·s/cm², which is not directly measured but the reactor configuration and lamp specifications from the manufacturer (Philips UV-C output of approximately 21 W per lamp) were used to estimate the radiation flux. The stirrer was positioned between the reactor walls and the UV lamp system as shown in Fig. 2^14^.

For each experiment, one liter of cosmetic wastewater was filled in a quartz cylinder batch reactor. The pH was adjust using sulfuric acid, followed by addition of the required dosage of iron salts, and finally hydrogen peroxide^15^. The reaction was initiated by switching UV lamps. Stirrer was used to assure that reactants were completely blended. All experiments were conducted at ambient temperature (25 ± 2 °C), and temperature was monitored periodically using a digital thermometer to ensure thermal stability. After the reaction time elapsed, a small dose of NaOH was added immediately to each sample to quench the reaction by decomposing residual hydrogen peroxide and raising the pH to inhibit further radical generation. The samples were then left to stabilize for a few minutes before COD analysis. Although NaOH quenching may not fully eliminate all residual H₂O₂, care was taken to minimize interference by standardizing the quenching volume and timing across all samples. In addition, COD values were verified through duplicate measurements to ensure consistency and accuracy.

### Analytical methods

Chemical oxygen demand (COD) was measured using the closed reflux colorimetric method with a HANNA Instruments HI83314 photometer, following standard procedures outlined in Standard Methods for the Examination of Water and Wastewater. Samples were filtered through 0.45 μm syringe filters before analysis to remove suspended solids and ensure accuracy.

Biochemical oxygen demand (BOD₅) was determined using the standard five days incubation method at 20 ± 1 °C and DO concentrations were measured using a portable DO meter (HANNA HI5421) method equipped with polarographic probe.

pH was monitored before and after treatment using a digital pH meter (Hanna HI2211). All glassware was washed with distilled water and dried before use. Calibration of instruments was performed prior to measurements to ensure reliability.

The biodegradability index (BOD₅/COD) was calculated to evaluate the enhancement in wastewater treatability after each AOP treatment.

### Statistical analysis

All experiments were conducted in triplicate, and the mean values were used for statistical evaluation. A multiple linear regression model was developed using IBM SPSS Statistics (version 25) to assess the individual and combined effects of four independent variables: pH, H₂O₂ dose (mL/L), Fe²⁺ or Fe³⁺ concentration (g/L), and UV irradiation time (min), on the COD removal efficiency (%).

The regression model was tested for statistical significance using analysis of variance (ANOVA), with a confidence level of 95% (*p* < 0.05). The model’s predictive accuracy was evaluated using the coefficient of determination (R²), adjusted R², and the standard error of estimate (SEE). Residual plots and normality tests were also used to validate the model assumptions and goodness of fit.

The resulting regression equation was then used to simulate COD removal under various operational conditions, allowing a deeper understanding of the relative importance of each parameter and the optimization of the treatment process.

## Results and discussion

### Photo-chemical oxidation

The cosmetic wastewater used in this research, was subjected for the treatment with different types of the photo-degradation such as UV radiation only, UV/H_2_O_2_, photo Fenton and photo Fenton like reaction. Different parameters were also investigated to define the optimum operating conditions in each process.

The potential interference of residual hydrogen peroxide in COD analysis was considered, and a standardized quenching step using NaOH was implemented. Although slight underestimation is possible, COD measurements were performed in duplicates to ensure the consistency and observed trends across all experimental runs indicate reliable comparative results.

The OFAT approach was selected to allow for direct assessment of each variable’s individual effect, considering the complexity and variability of the real wastewater matrix. Although DOE could provide further optimization^16–18^it was beyond the scope of the current exploratory study and will be considered in future work.

#### Direct photolysis (UV irradiation only)

UV radiation can affect the rate at which organic materials in water decompose in two ways. Firstly, it combined with hydrogen peroxide and promoted the formation of radicals. Secondly, when the radiation wavelength corresponds to the UV spectrum of the substance, it starts degraded, it permits direct photolysis to take place. When water absorbs UV photons, it breaks into the H atom and the OH radical, which react quickly with organic solutes as shown in Eq. 1^19^

1 $${{\text{H}}_2}{{\text{O}}_{(l)}}+{\text{h}}\upnu \to {\text{O}}{{\text{H}}^ \bullet }+{{\text{H}}^+}$$

In presence of oxygen, the absorption of UV light by water is thought to start chain reactions. that produce hydroxyl and hydroxyl radicals. Only when the contaminants have high enough molar absorption coefficients, UV light may be used without any oxidants^20^.

##### Effect of irradiation time

**Fig. 3:**
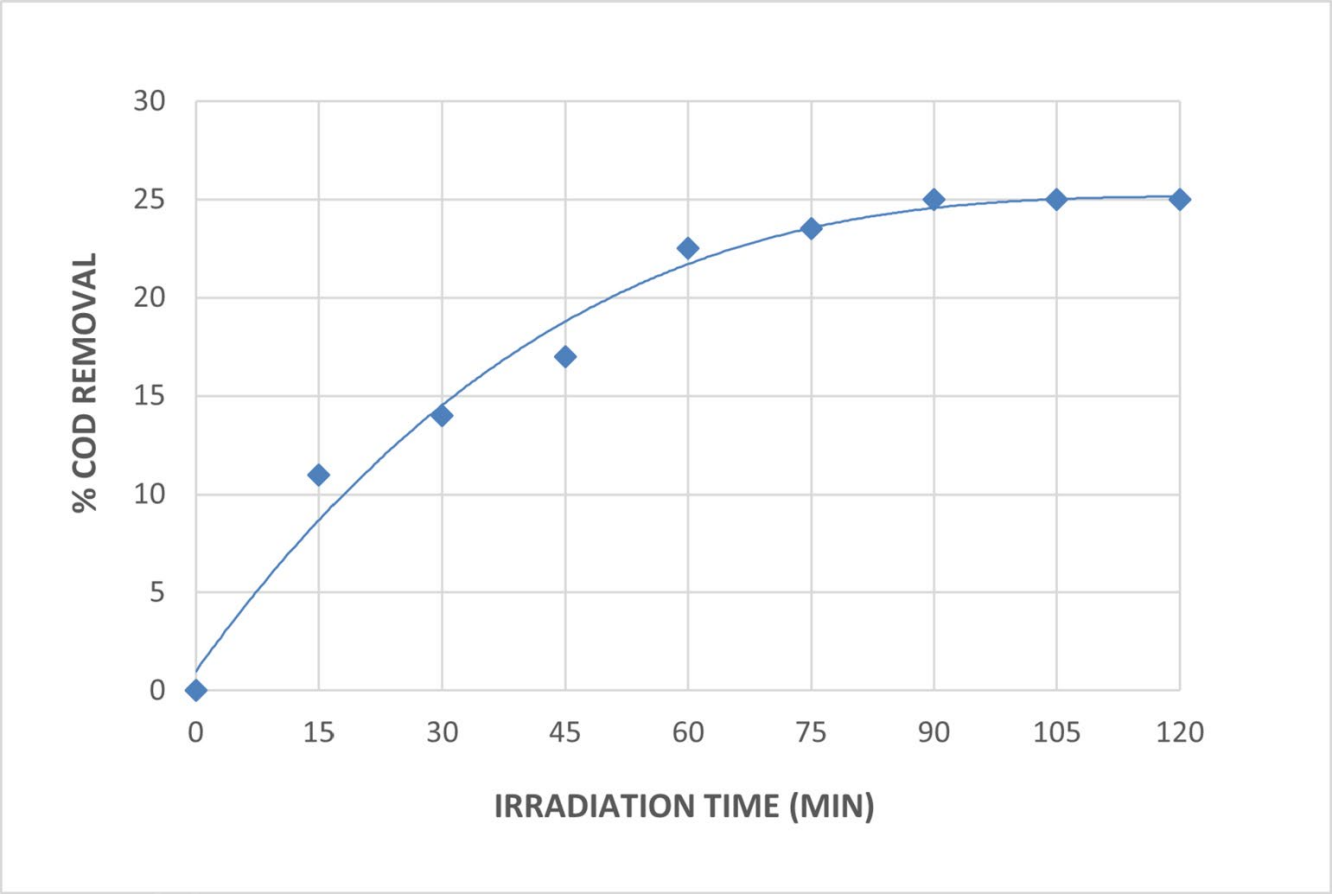
Effect of Irradiation Time on The Direct Photolysis of The Wastewater [pH = 7].

The irradiation duration effect on wastewater treatment utilizing direct photolysis at a pH of 7 is depicted in Fig. 3. The figure shows that as the irradiation period increased, the wastewater’s degradation efficiency with COD removal gradually increased. The percentage of COD removed within the 90-minute irradiation period was 25. There was no increase in the percentage of COD elimination as the irradiation period increased.

##### Effect of pH value

**Fig. 4:**
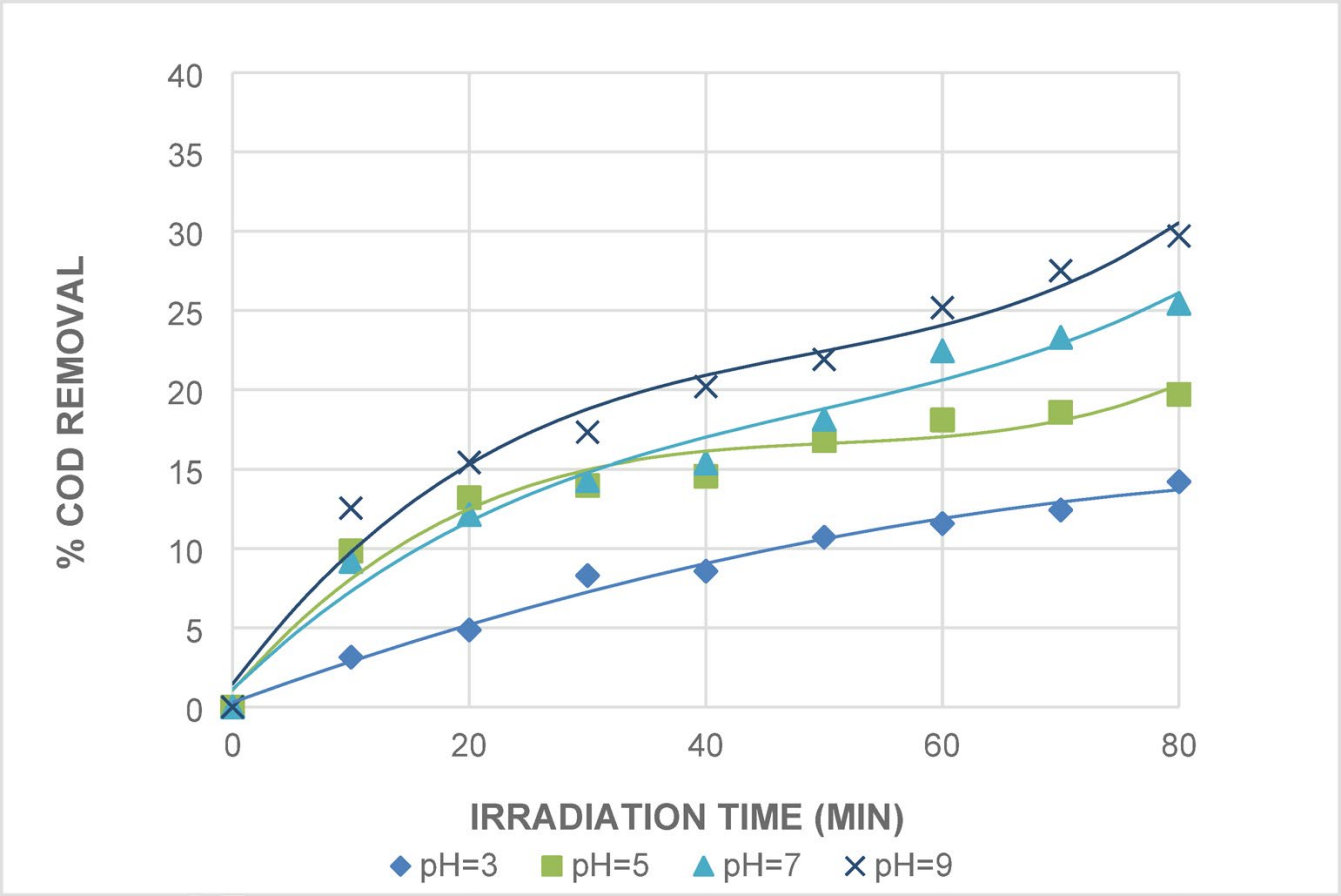
Effect of pH Values on The Direct Photolysis of Wastewater [Time = 80 Min].

 The pH is a crucial parameter to consider. The impact of pH on the wastewater’s direct photolysis was investigated. Figure 4 displays the percentage of COD removed in relation to various pH and irradiation time values. The results demonstrate that the percentage of COD removed by UV alone decreases from the alkali medium at pH = 9 to the acidic medium at pH = 3. The best outcomes were at pH values of 7 and 9, where COD removals were 25 and 29.6 at 80 min of irradiation. This suggests that OH radicals are produced at high pH levels during direct photolysis. At lower pH values, the prevailing process during irradiation was shown to be direct photolysis.

In literature, UV process for degradation of cosmetics wastewater wasn’t clearly investigated but B. Tawabini et al., 2004, studied the use of UV/H_2_O_2_ and direct photolysis to remove phthalates from contaminated water. Dimethyl phthalate (DMP) was utilized as a model compound, with a starting concentration of 20 ppm. The radiation was generated by a low-pressure mercury UV lamp with an intensity of 100 mWatt. The results demonstrated that, during an hour of exposure, over 60% of DMP were directly eliminated through UV light-induced activation. However, When the UV is applied to cosmetics wastewater, which contain several complex compounds including DMP, the percentage of COD removal decreased. This can be explained by the difficulty of breaking the intricate bonds between the contaminant’s molecules in cosmetics wastewater because they can’t absorb UV light at all. Therefore, we will need to utilize oxidants^21^.

##### Effect of addition of catalyst

**Fig. 5:**
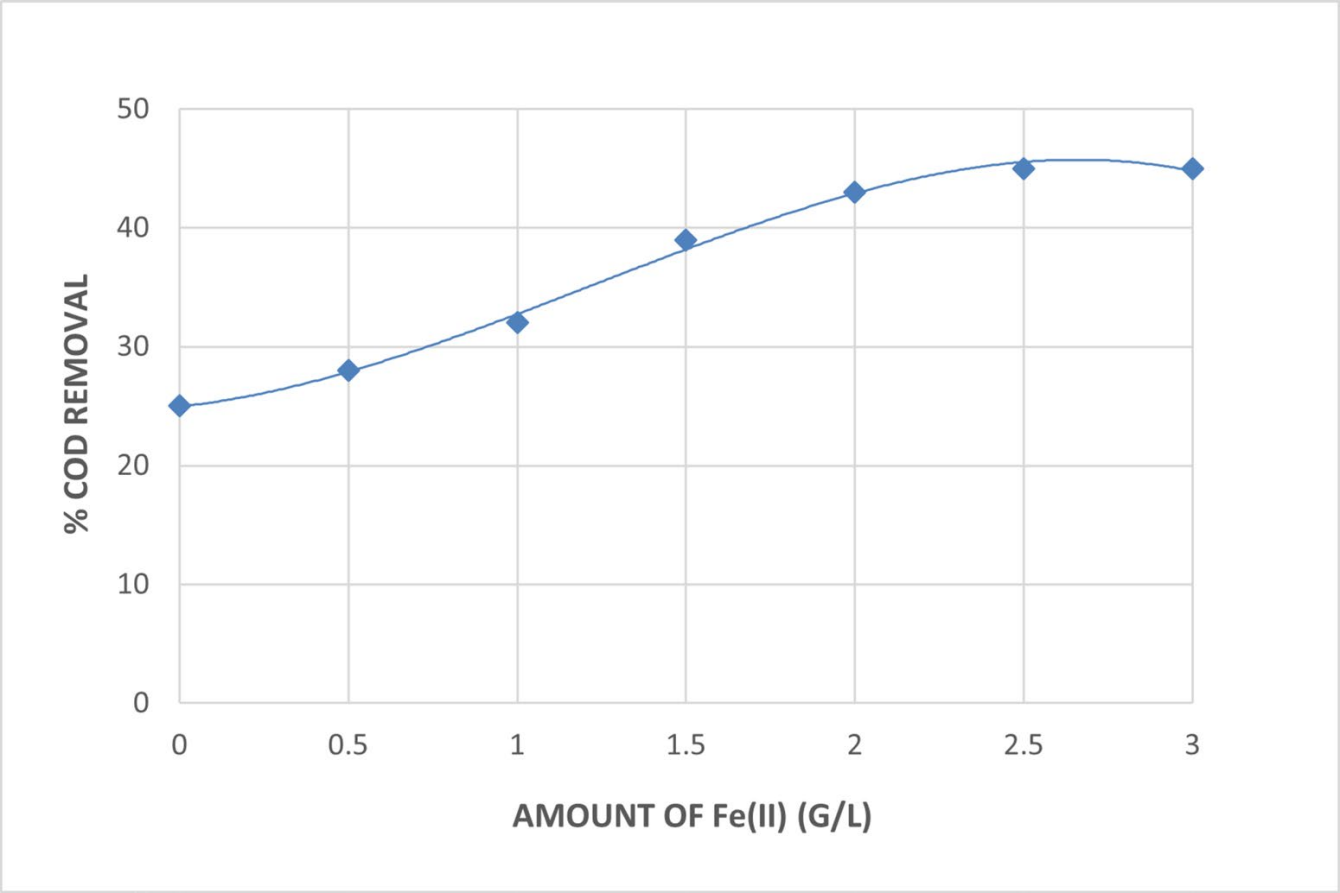
Effect of Addition of Fe^+2^ as Photocatalyst on The Direct Photolysis of The Wastewater [pH = 7, Irradiation Time = 90 Min].

The direct photolysis process was started for the treatment of the wastewater collected for this research by examining the impact of adding Fe^+2^ as a photocatalyst in the form of ferrous sulphate heptahydrated (FeSO_4_.7H_2_O)^22^. At a pH of 7 and after 90 min of irradiation, Fig. 5 illustrates the impact of the amount of Fe^+2^ used as a photocatalyst on the removal of COD. As seen in Fig. 5, the inclusion of Fe^+2^ as a photocatalyst results in a minor improvement in COD removal; however, no additional increase in COD removal was observed behind 3 g/L Fe^+2^. This is explained by the rise in the number of free hydroxyl radicals generated.

#### UV/hydrogen peroxide system

Many compounds have very low molar absorption coefficients or do not absorb UV light at all. Under such conditions, H_2_O_2_ may be added to encourage degradation. When the pollutant solution containing H_2_O_2_ is exposed to UV radiation with wavelengths shorter than 280 nm, the UV/H_2_O_2_ process is impacted. Hemolytic cleavage of H_2_O_2_ results from this. Equation 2 states that hydroxyl radicals are produced during this type of treatment. Two hydroxyl radicals are produced when it photodegrades^23^.

2 $${{\text{H}}_2}{{\text{O}}_{2({\text{aq}})}}+{\text{h}}\upnu \to 2{\text{O}}{{\text{H}}^ \bullet }$$

The efficiency of organic waste degradation is significantly increased when UV and H_2_O_2_ are combined. In contrast to photocatalysis, homogeneous photolysis techniques do not require a solid catalyst separation process following treatment. Short wavelengths of intense UV light and additional chemical oxidants like ozone and hydrogen peroxide are necessary for photolysis to be effective^24^.

##### Effect of irradiation time

**Fig. 6:**
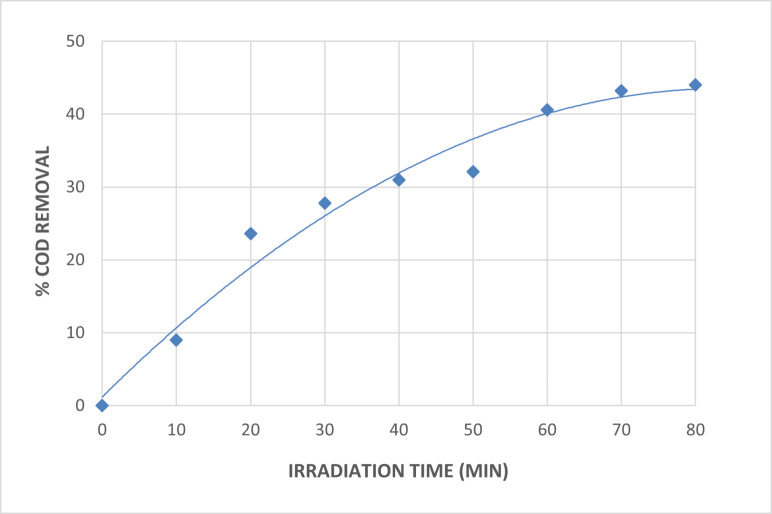
Effect of Irradiation Time on UV/H_2_O_2_ Process for The Treatment of The Wastewater [pH = 7, H_2_O_2_ = 3mL/L and Time = 80 Min].

The impact of irradiation duration on wastewater treatment using the UV/H_2_O_2_ process at a neutral pH of 7 and a hydrogen peroxide dosage of 3 mL/L is displayed in Fig. 6. Figure 6 shows that the longer the irradiation period, the higher the degradation efficiency of the wastewater with COD removal. The percentage of COD removed within the 80-minute irradiation period was 44. The percentage of COD removal did not increase as the irradiation period increased.

##### Effect of the amount of H_2_O_2_

**Fig. 7:**
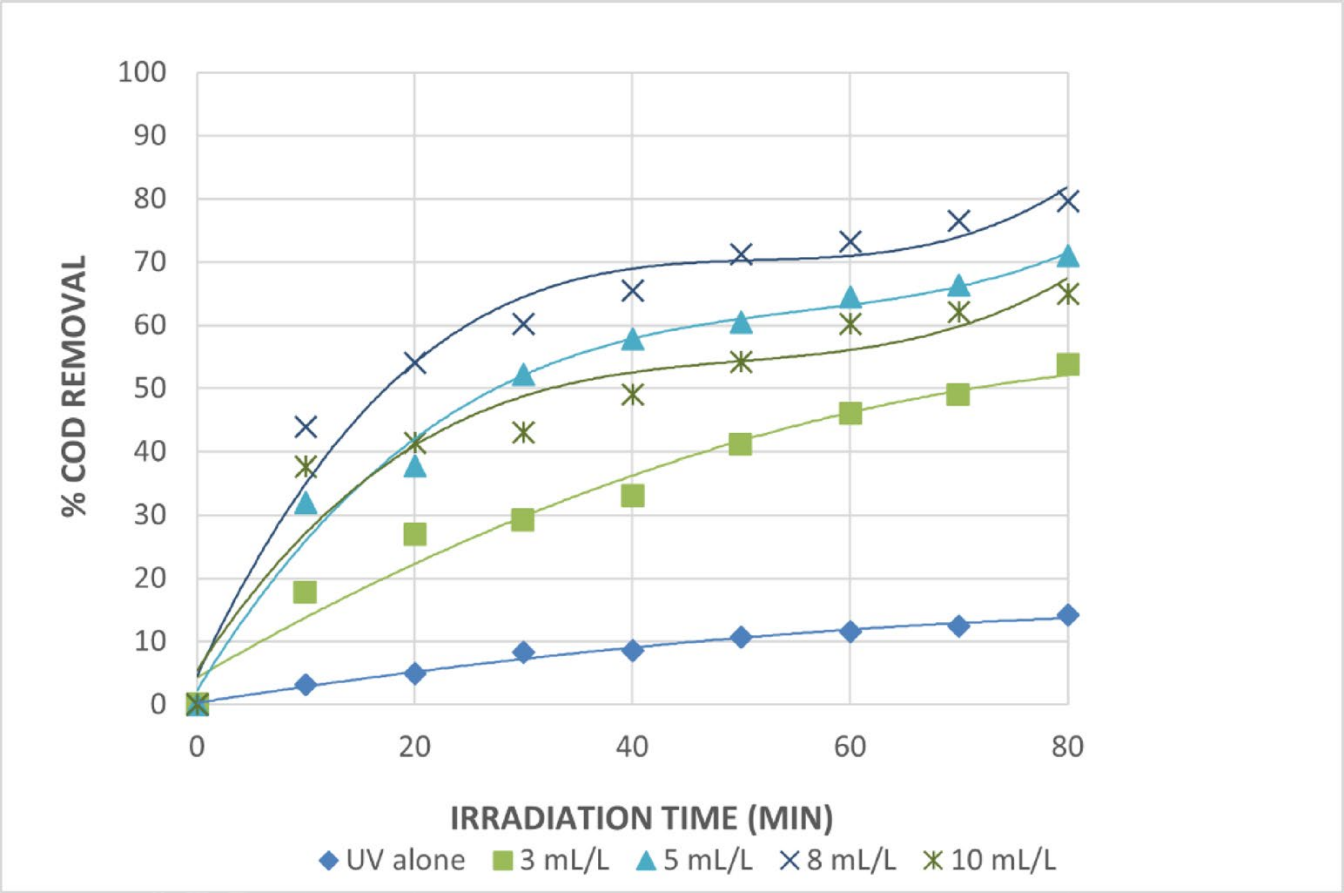
Effect of Addition an Amount of H_2_O_2_ Photocatalyst on The Treatment of The Wastewater by UV/H_2_O_2_ Process. [pH = 3 and Time = 80 Min].

When hydrogen peroxide was coupled with UV irradiation, the rate of COD removal increased substantially in comparison to direct photolysis, even though hydrogen peroxide did not oxidize the organic wastes included in the wastewater collected for this research. The percentage of COD removed as a function of irradiation time at various H_2_O_2_ input dosages at pH = 3 is shown in Fig. 7. When organic wastes were photolyzed without H_2_O_2_, the results were rather moderate, and the organic components degraded slowly. When hydrogen peroxide concentration increased, the percentage removal of COD first increased with the addition of H_2_O_2_. However, peroxide has a detrimental influence on the rate of degradation. Figure 7 shows that in a direct photolysis investigate, the percentage of COD removed at 60 min was 11.57%, and after 80 min of irradiation, it was 14.21%.

Hydroxyl radicals produced during the photolysis of hydrogen peroxide were the primary species responsible for the removal of COD in this process when the photolysis was conducted with an initial hydrogen peroxide concentration of 3 mL/L. The COD removal rate increased from 14.21% in the absence of H_2_O_2_ concentration to 53.67%. However, adding more hydrogen peroxide to the initial amount interacts with these radicals and so inhibits COD degradation. Figure 7 indicates that the optimal initial hydrogen peroxide addition quantities were 5 and 8 milliliters per liter, with COD removal percentages of 70.9 and 79.7 after 80 min of irradiation. Since there is a slight increase in COD removal with 8 mL/L of hydrogen peroxide, 5 mL/L was chosen from an economic standpoint. The COD degradation rate decreased to 64.94% when the initial hydrogen peroxide addition exceeded 8 mL/L because the hydrogen peroxide concentration was increased, and its hydroxyl radical scavenging effect became significant.

##### Effect of pH

**Fig. 8:**
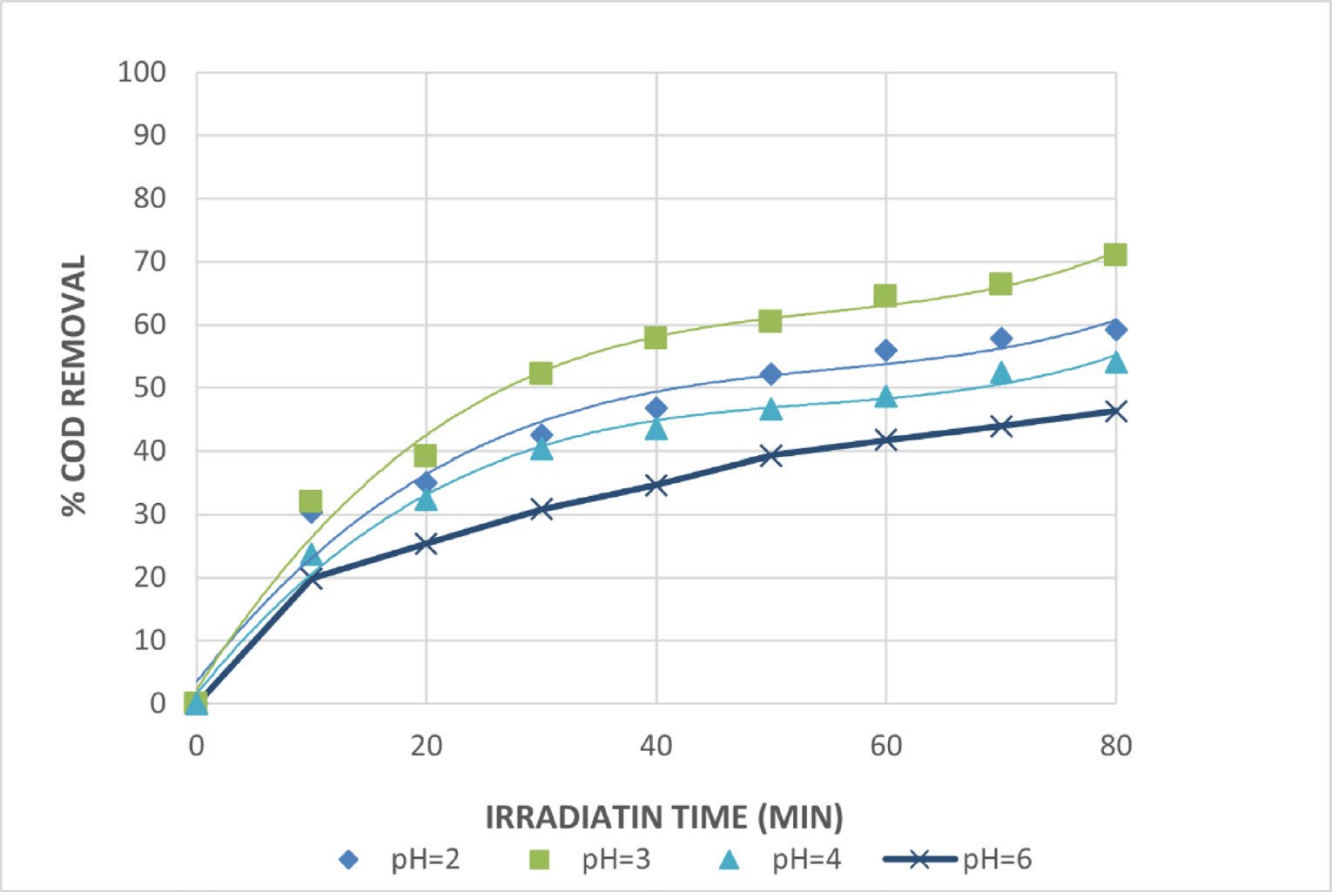
Effect of pH Values on The Treatment of The Wastewater by UV/H_2_O_2_ Process. [H_2_O_2_ = 5mL/L and Time = 80 Min].

Compared to direct photolysis, the UV/H_2_O_2_ system decreases considerably the rate at which organic materials decompose in basic media. Since a higher efficiency of oxidation of the organics contained in water was achieved by employing the UV/H_2_O_2_ combination at low pH, this suggests that H_2_O_2_ is more sensitive and effective in acidic conditions. The impact of pH value on COD removal efficiency is depicted in Fig. 8. Figure 8 illustrates that, over an 80-minute irradiation period, the percentage of COD removal increased from pH = 2 to pH = 3, with removal rates of 59.2 and 70.9, respectively. The percentage of COD removals reduced from 54 at pH values of 4 and 6 to 46.3 at pH values greater than 3. As a result, the optimum pH is 3 and the COD removal percentage is 70.9 after 80 min of irradiation.

Previous research focused on using UV/H_2_O_2_ for industrial wastewater treatment. B. Tawabini et al., 2004, showed that UV/H_2_O_2_ processes may remove dimethyl phthalate from contaminated water at an initial concentration of 20 ppm. When the DMP-spiked water was first treated with H2O2 before being exposed to UV light (i.e., UV/H_2_O_2_), the removal efficiency enhanced. After 45 min, over 98% of the DMP was removed. On the other hand, the percentage of COD removal dropped when UV/H_2_O_2_ was applied to wastewater from cosmetics, which contains complex chemicals with different types of phthalates including DMP^21^.

G. Sudarjanto et al., 2005, studied the use of UV/H_2_O_2_ technology to photo oxidize a reactive azo dye from the textile industry. A low-pressure mercury arc lamp (60 W) producing at 253.7 nm was the source of the UV light. After only 20 to 30 min of irradiation, complete decolorization was attained. Nevertheless, almost 60% of the COD remained after the active dye had been almost entirely removed from the solution, indicating that the dye molecule had only partially broken down. In our research, when applying this process on cosmetics wastewater, it was found that the percentage of COD is increasing from 40 to 70.9% with increasing of irradiation time to 80 min^25^.

According to the results, the UV/H_2_O_2_ process generally showed good promise for eliminating contaminants from industrial wastewater; however, more treatment may be required to fully mineralize the organic intermediates.

#### Photo-Fenton reaction and photo-Fenton-like reaction

The Photo-Fenton and Photo-Fenton-like processes rely on the generation of hydroxyl radicals (•OH) through reactions between iron salts and hydrogen peroxide under UV irradiation. In the Photo-Fenton system, Fe^+2^ reacts with H₂O₂ to produce Fe^+3^ and •OH as shown in Eq. 3.

3 $${\text{F}}{{\text{e}}^{+\,{\text{2}}}}_{{({\text{aq}})}}\,+\,{{\text{H}}_{\text{2}}}{{\text{O}}_{{\text{2(aq)}}}} \to {\text{ F}}{{\text{e}}^{+\,{\text{3}}}}_{{({\text{aq}})}}\,+\,{\text{O}}{{\text{H}}^--}_{{({\text{aq}})}}\,+\,{\text{O}}{{\text{H}}^ \bullet }$$

The generated Fe^+3^ can be photochemically reduced back to Fe^+2^ under UV light (λ < 400 nm), allowing continuous radical production as shown in Eq. 4.

4 $${\text{F}}{{\text{e}}^{+\,{\text{3}}}}_{{({\text{aq}})}}\,+\,{\text{h}}\upnu \to {\text{ F}}{{\text{e}}^{+\,{\text{2}}}}_{{({\text{aq}})}}\,+\,{\text{O}}{{\text{H}}^ \bullet }$$

In the Photo-Fenton-like process, Fe^+3^ is the starting catalyst and reacts with H₂O₂ to generate Fe^+2^ and hydroperoxyl radicals (HO₂•), which can further participate in oxidation reactions as shown in Eq. 5.

5 $${\text{F}}{{\text{e}}^{+\,{\text{3}}}}_{{({\text{aq}})}}\,+\,{{\text{H}}_{\text{2}}}{{\text{O}}_{\text{2}}}_{{({\text{aq}})}} \to {\text{ F}}{{\text{e}}^{+\,{\text{2}}}}_{{({\text{aq}})}}\,+\,{\text{H}}{{\text{O}}_{\text{2}}}^{ \bullet }+{\text{ }}{{\text{H}}^+}$$

UV irradiation enhances this process by accelerating the regeneration of Fe^+2^ and increasing the yield of hydroxyl radicals. However, excessive concentrations of iron or H₂O₂ can lead to radical scavenging and reduced efficiency. Therefore, optimizing pH, iron dosage, and H₂O₂ concentration is crucial for maximizing degradation efficiency in both systems^26,27^.

##### Effect of irradiation time

**Fig. 9:**
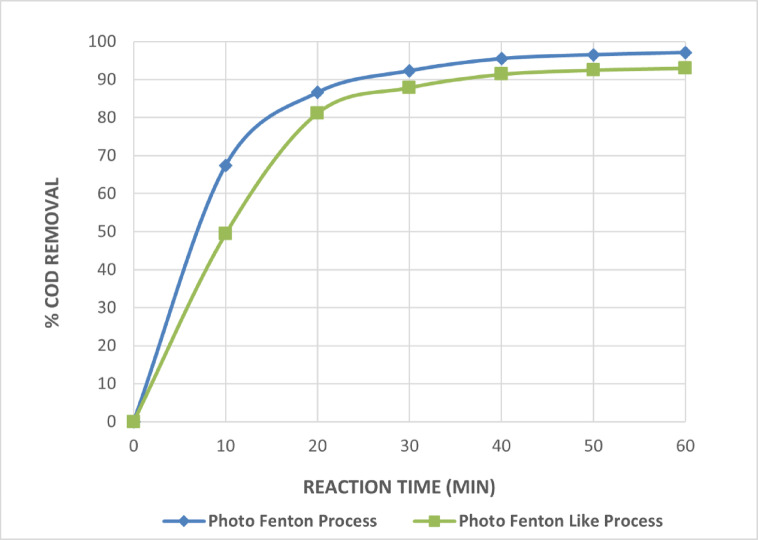
Effect of Irradiation Time on Photo Fenton and Photo Fenton Like Processes for The Treatment of Wastewater. [pH = 3,Fe^+2^=0.75 g/L, H_2_O_2_ = 1mL/L and Time = 60Min].

The ideal irradiation duration for both photo Fenton and photo Fenton-like was 40 min at pH equal to 3, according to Fig. 9. The initial hydrogen peroxide concentration was 1 mL/L, and the initial Fe^+2^ and Fe^+3^ concentrations were 0.75 g/L for both photo Fenton and photo Fenton-like.

##### Effect of the pH value

**Fig. 10:**
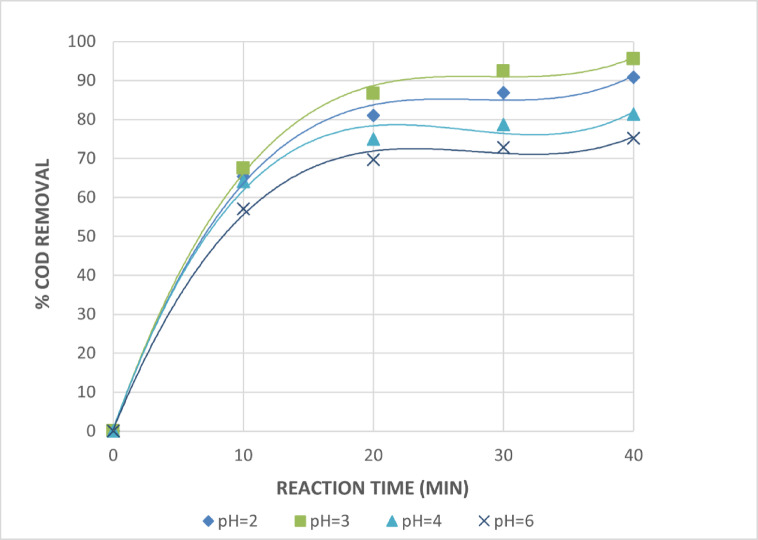
Effect of pH Values on The Treatment of The Wastewater by Photo Fenton Reaction. [H_2_O_2_ = 1mL/L, Fe^+2^=0.75 g/L and Time = 40 Min].

**Fig. 11:**
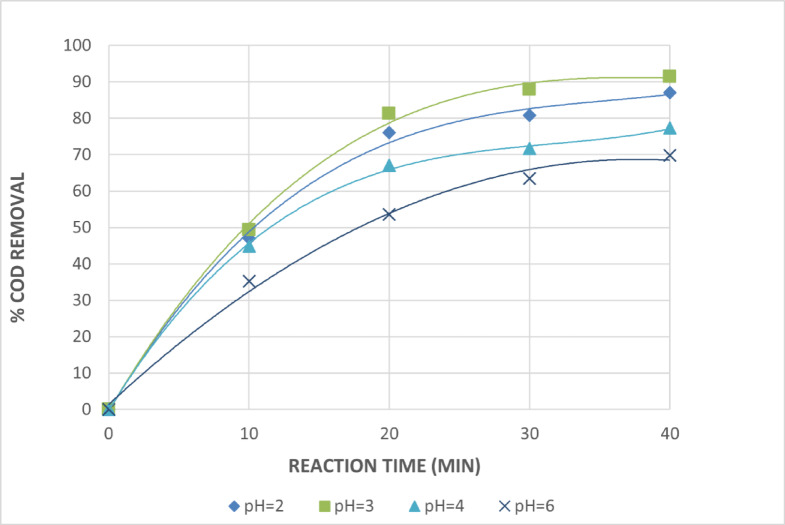
Effect of pH Values on The Treatment of The Wastewater by Photo Fenton Like Reaction. [H_2_O_2_ = 1mL/L, Fe^+3^ =0.75 g/L and Time = 40 Min].

Because the oxidation potential is proportional to the pH value, the pH value has a significant impact on the oxidation potential of OH• radicals (E^0^ = 2.8 V and E^14^ = 1.95 V). Furthermore, pH has a significant impact on inorganic carbon concentration and the hydrolytic speciation of Fe^+3^ species^28^.Therefore, it is necessary to determine how pH functions in the photo-assisted Fenton reaction. The photo-Fenton and photo-Fenton-like systems are most active when the pH is between 2.8 and 3. The generation of OH• radicals and, consequently, the oxidation efficiency is influenced by the pH value. The degradation is significantly reduced at pH values above 6 because iron precipitates as a hydroxide derivative, which lowers the availability of Fe^+2^ and the transmission of radiation. The dissociation and auto-decomposition of H_2_O_2_ are additional factors contributing to the ineffective removal at pH > 3^29–31^. Figures 10 and 11 demonstrate the significant influence of pH on COD removal efficiency using the photo-Fenton and photo-Fenton-like processes, respectively. The efficiency increased markedly at acidic pH, with optimal removal observed at pH = 3. The photo Fenton and photo Fenton like processes produced maximal COD removals of 95.5 and 91.4%, respectively, at pH = 3 and after 40 min of irradiation. The COD removals for both the photo Fenton and photo Fenton like processes significantly reduce to 75.2% and 69.7%, respectively, at a pH of 6. This is because iron precipitates as hydroxide at higher pH values, which decreases radiation transmission.

##### Effect of initial hydrogen peroxide concentration

**Fig. 12:**
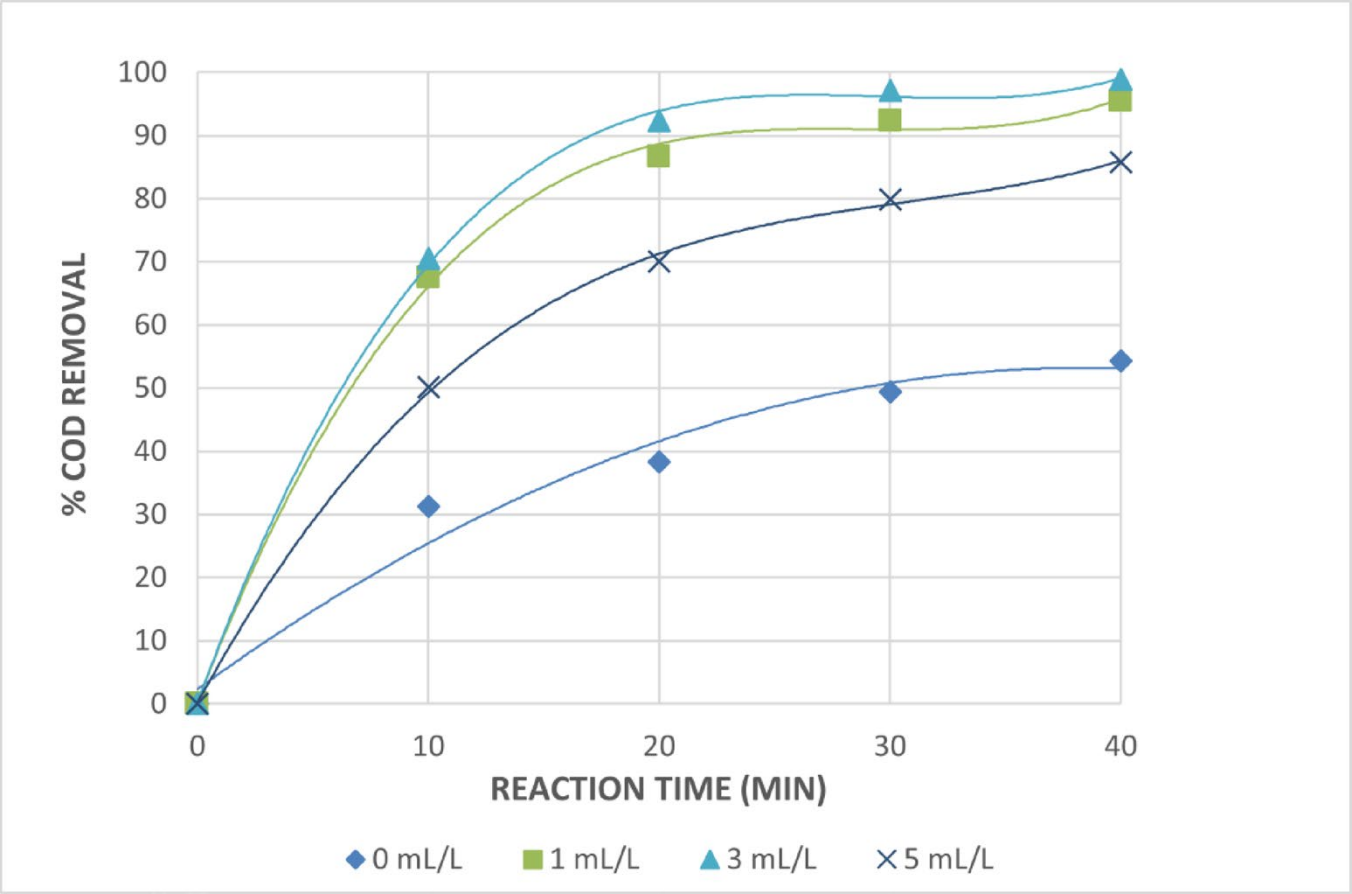
Effect of Initial Amount of H_2_O_2_ on The Treatment of The Wastewater by Photo Fenton Reaction. [pH = 3, Fe^+2^=0.75 g/L and Time = 40 Min].

**Fig. 13:**
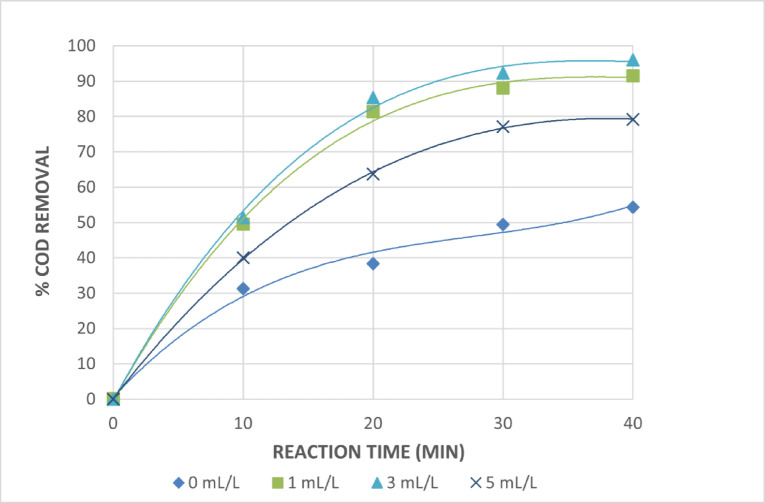
Effect of Initial Amount of H_2_O_2_ on The Treatment of The Wastewater by Photo Fenton Like Reaction. [pH = 3, Fe^+3^ =0.75 g/L and Time = 40 Min].

Several experiments were conducted by varying the initial H_2_O_2_ concentrations at constant initial pH 3, initial Fe^+2^ and Fe^+3^ of 0.75 g/L for the photo Fenton and photo Fenton like systems, and within irradiation time of 40 min in order to determine the role of H_2_O_2_ concentration on the photo-catalytic degradation of cosmetic wastewater taken in this investigation in the photo Fenton and photo Fenton-like systems. Figures 12 and 13 illustrate how the degrading efficiency, as indicated by the percentage of COD removals, increases as the H_2_O_2_ concentration rises from 0 to 3 mL/L. This phenomenon is explained by the impact of the more OH^•^ radicals that are created. However, as the hydrogen peroxide concentration increases through this point, the reaction rate levels out and occasionally suffer from adverse effects. This could be the result of OH^•^ radical recombination and the auto-decomposition of H_2_O_2_ to oxygen and water as shown in Eq. 6and Eq. 7. Because H_2_O_2_ itself increases the OH^•^ radicals scavenging capacity, too much of it will react with OH^•^, competing with organic contaminants and lowering treatment efficiency. However, because auto-scavenging processes are more likely to occur at greater H_2_O_2_ concentrations, a higher dose of H_2_O_2_ also results in a higher generation of hydroxyl radicals. For the best degradation, H_2_O_2_ should be injected at the ideal concentration^32^.

6 $${\text{2}}{{\text{H}}_{\text{2}}}{{\text{O}}_{{\text{2(aq)}}}} \to {\text{ 2}}{{\text{H}}_{\text{2}}}{{\text{O}}_{({\text{l}})}}+{\text{ }}{{\text{O}}_{\text{2}}}_{{({\text{g}})}}$$

7 $${\text{O}}{{\text{H}}^ \bullet }+{\text{ }}{{\text{H}}_{\text{2}}}{{\text{O}}_{{\text{2}}({\text{aq}})}} \to {\text{ H}}{{\text{O}}_{\text{2}}}^{ \bullet }+{\text{ }}{{\text{H}}_{\text{2}}}{{\text{O}}_{({\text{l}})}}$$

The dose of 1 mL/L H_2_O_2_ is thought to be the ideal dose from an economic perspective because there is a very slight increase in the percentage of COD removals at initial hydrogen peroxide concentrations of 1 and 3 mL/L. After 40 min of irradiation, it is discovered that the ideal H_2_O_2_ concentration for treating the wastewater under investigation is 1 mL/L, with 95.5 and 91.4% COD reductions for photo Fenton and photo Fenton like processes, respectively.

##### Effect of initial amount of Fe ion

As a photo-catalyst, iron in both its ferrous and ferric forms need a pH lower than 4. The experiment was conducted using varied amounts of the iron salt at varying constant initial concentrations of hydrogen peroxide to determine the ideal Fe^+2^ or Fe^+3^ amounts.

**Fig. 14:**
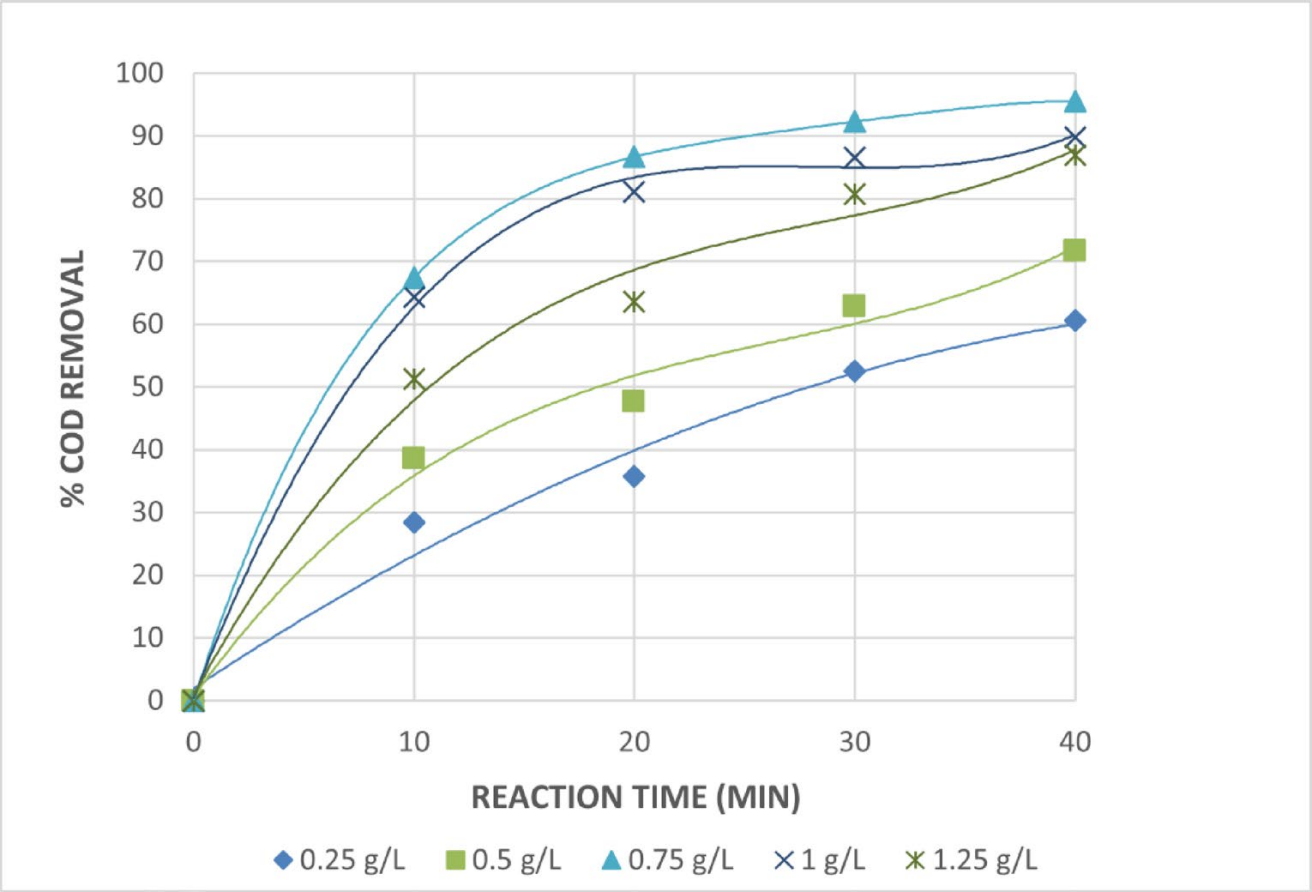
Effect of Initial Amount of Fe^+2^on The Treatment of The Wastewater by Photo Fenton Reaction at Constant Initial Amount of H_2_O_2_ = 1 mL/L. [pH = 3 and Time = 40 Min].

**Fig. 15:**
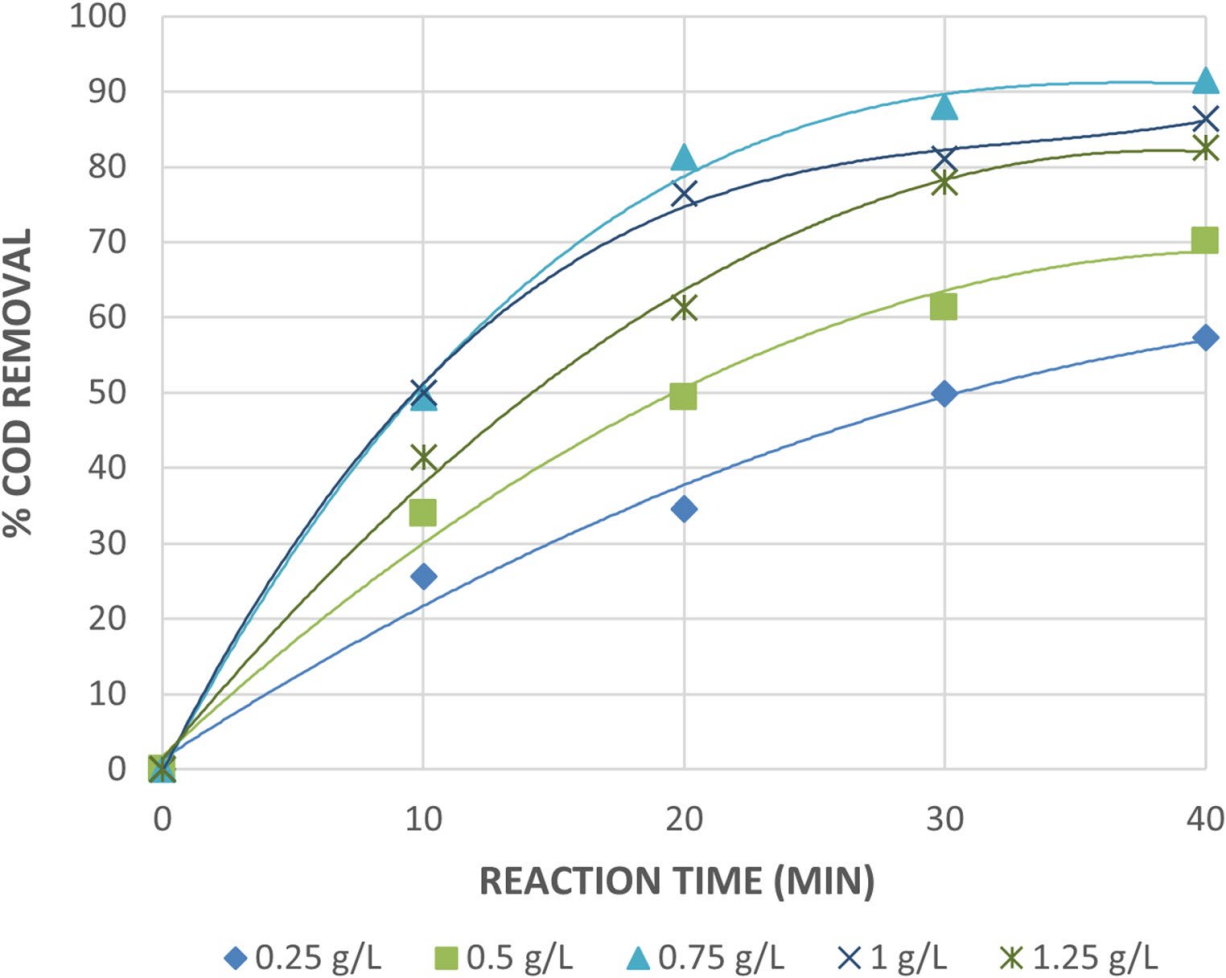
Effect of Initial Amount of Fe^+3^ on The Treatment of The wastewater by Photo Fenton Like Reaction at Constant Initial Amount of H_2_O_2_ = 1 mL/L. [pH = 3 and Time = 40Min].

The effect of Fe^+2^ concentration on wastewater treatment by photo Fenton reaction against reaction time under constant H_2_O_2_ value of 1mL/L is shown in Fig. 14. The effect of Fe^+3^ concentration on wastewater treatment by photo Fenton like reaction vs. reaction time under constant H_2_O_2_ value of 1mL/L is shown in Fig. 15.

According to the figures, the effectiveness of photo Fenton and photo Fenton like for COD degradation was increased by adding either Fe^+2^ or Fe^+3^ at all initial H_2_O_2_ concentrations. When iron salt is added to 0.75 g/L Fe^+2^ or Fe^+3^ at pH equal to 3, 1 mL/L H_2_O_2_, and after 40 min of irradiation, the COD removals increase to 95.5 and 91.4% for photo Fenton and photo Fenton like processes, respectively. Under the same conditions, the COD removals decrease when the dose of either Fe^+2^ or Fe^+3^ is raised over 0.75 g/L. The COD removals for the photo Fenton and photo Fenton like processes were 89.8% and 86.4% at 1 g/L of either Fe^+2^ or Fe^+3^, respectively. At the same H_2_O_2_ doses, the same trend was seen; however, for both processes, a decrease in COD removals was noted when the initial H_2_O_2_ was 5 mL/L.

The degradation was unaffected in every instance by adding more than 0.75 g/L of Fe^+2^ or Fe^+3^. However, a higher addition of iron salt produced a muddy appearance that prevented the absorption of UV light needed for photolysis and led to the recombination of OH radicals. Here, Fe^+2^ or Fe^+3^ works as a scavenger by reacting with OH radicals^33,34^.

The ratio of H_2_O_2_ to Fe (II) or Fe (III) should be as low as possible to prevent recombination and reduce the amount of sludge produced by the iron complex.

Many studies have been conducted to study the applying of photo Fenton process on industrial wastewater. This research agreed with Ebrahiem E. Ebrahiem et al.,2017 that studied the removal of organic pollutants from cosmetics wastewater using photo Fenton process under the optimal conditions (pH = 3, 1mL/L H_2_O_2_, 0.75 g/L Fe^+2^ and 40 min reaction time), the process achieved over 95% COD removal. This can be explained by the addition of UV light to the Fenton process (photo-Fenton) enhanced pollutant removal by approximately 20% compared to the traditional Fenton reaction^35^.

The results indicates that photo-Fenton oxidation is a highly efficient and recommended method for the treatment of industrial wastewater containing organic pollutants, particularly before biological treatment.

#### pH variation during optimal treatment conditions

In this research, multiple pH values were examined for each treatment process to determine the optimal pH for maximum COD removal. Once the optimal pH was identified for each system, the corresponding experiments were used to monitor pH variation before and after treatment. The initial pH was adjusted using sulfuric acid, and the final pH was recorded immediately after the reaction ended.

The pH remained relatively stable in UV and UV/H₂O₂ systems (fluctuations < 0.3 units). However, in the Photo-Fenton and Photo-Fenton-like processes, a slight decrease in pH (from 3.0 to approximately 2.5–2.7) was observed due to the generation of acidic oxidation intermediates and consumption of hydroxyl ions. These changes are consistent with the expected behavior of iron catalyzed AOPs and were considered when evaluating treatment performance.

## Kinetic reactions

It is extremely challenging to perform a kinetic analysis of photo oxidation based on the evolution of individual components due to the complicated characteristics of these wastewaters. Additionally, because of the wide variations in chemical composition of various wastewaters, such an approach would be of limited use. To determine the process’s kinetics from a broad perspective, a more useful criterion based on the evolution of COD has been adopted.

The experimental COD against time results from photo oxidation runs performed under ideal operational conditions for UV, UV/H_2_O_2_, photo Fenton process, and photo Fenton-like process were used to conduct the kinetic study. In UV alone, the optimum condition was pH = 9 at 80 min irradiation time. For UV/H_2_O_2,_ the optimum operation conditions where Hydrogen Peroxide concentration was 5 mL/L within 80 min irradiation time. The optimum irradiation time was 40 min for both photo Fenton and photo Fenton-like at pH equals 3, initial amount of hydrogen peroxide equals 1 mL/L and initial amounts of Fe^+2^ and Fe^+3^ equal 0.75 g/L for the both photo Fenton and photo Fenton-like.

**Fig. 16:**
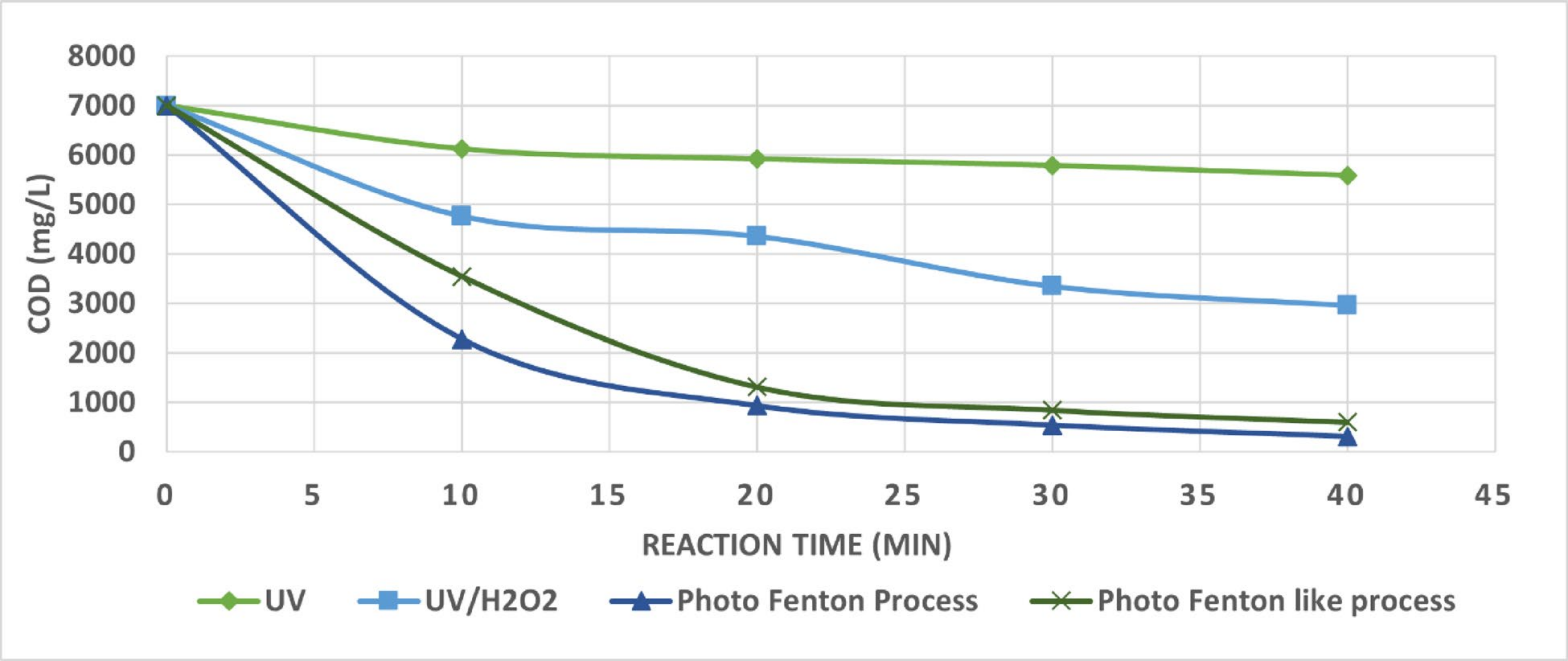
Evolution of COD (mg/L) Conversion Against Reaction Time for The Photo Oxidation Processes at Optimum Operating Conditions.

**Fig. 17:**
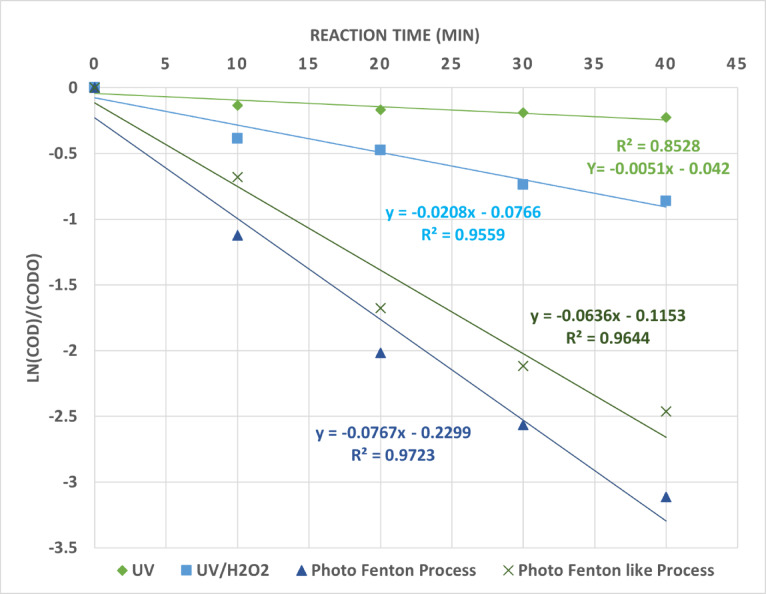
Pseudo-first-order plot for COD removal for photo Oxidation Processes at Optimum Operating Conditions.

Figure 16 shows evolution of COD against reaction time for UV, UV/H_2_O_2_, photo Fenton process, and photo Fenton-like process. The rapid and high generation of hydroxyl radicals in the photo Fenton process cast illustrates that the COD reduction occurs more quickly with the photo Fenton process than with other methods. A pseudo-first order kinetic equation of the kind is well suited to the experimental results shown in Fig. 17. According to the following relationship, the degradation kinetics of the wastewater collected for this research utilizing the photo oxidation process can be represented by pseudo-first-order kinetics in Eq. 8^36^.

8 $${\text{Ln }}\left[ {{\text{COD}}} \right]/{\left[ {{\text{COD}}} \right]_0}={\text{ }} - {\text{k t}}$$

Where [COD]_0_ is initial the chemical oxygen demand of the organic substances, [COD] is the chemical oxygen demand of the organic substances at *t* times, and *k is* the expected pseudo-first-order rate constant.

The experimental findings are fitted to this equation in Fig. 17, which indicates the variations in the COD degradation rates of the wastewater collected during the photo oxidation processes. A linear relationship with the pseudo-first order is provided by this relation. The equations correlation factor values, which ranged from 0.85 to 0.97, ensure that this relationship is fit with the pseudo-first order. The entire reaction rate constant, k, is represented by the slop of this linear equation. At the optimum operating conditions, the values obtained for the apparent kinetic constants overall reaction rate constant (the pseudo-first order rate constant) were 0.0051, 0.0208, 0.0767 and 0.0636 for UV, UV/H_2_O_2_, photo Fenton process and photo Fenton like process respectively.

As can be seen, the rate constant is highest for the photo Fenton process indicating the fastest degradation of COD. The UV- only process has the slowest degradation rate.

## Biodegradability of the treated wastewater

Biodegradability is a critical parameter in evaluating the efficiency of wastewater treatment processes, particularly in industrial effluents like those from the cosmetics industry. The biodegradability index, expressed as the ratio of BOD₅ to COD, provides insights into the organic matter’s susceptibility to microbial degradation. In the studied cosmetic wastewater, the low BOD₅/COD ratio of 0.28 indicates poor biodegradability, suggesting the presence of complex organic pollutants resistant to biological treatment. Conventional biological processes often struggle to reduce the organic load effectively in such effluents. Advanced oxidation processes (AOPs), including UV, UV/H₂O₂, photo-Fenton and photo Fenton Like processes enhance biodegradability to 0.40, 0.66, 0.80, and 0.73, respectively, by breaking down complex pollutants into simpler, more bioavailable forms. By optimizing conditions such as pH, irradiation time, and chemical dosages, these methods can improve the biodegradability index, paving the way for integrated and cost-effective wastewater treatment approaches that meet stringent environmental regulations^37,38^.

As shown in Table 2, the biodegradability improvement is highest in the photo Fenton process, followed by the photo Fenton like process and UV/H₂O₂ process. The increase in BOD₅ after UV alone is attributed to the partial oxidation of large, recalcitrant molecules into smaller, more biodegradable intermediates such as short-chain organic acids and alcohols so, UV treatment alone results in a marginal increase in biodegradability.

**Table 2:** Biodegradability index for photo oxidation Processes.

Process	Final COD (mg/L)	Final BOD_5_ (mg/L)	Biodegradability (BOD_5_/COD)
Initial (Unreacted)	7000	1960	0.28
UV	4928	1969	0.40
UV/H_2_O_2_	2037	1346	0.66
Photo-Fenton	315	254	0.80
Photo Fenton Like	601	439	0.73

## Economic assessment

### Specific energy consumption

The efficiency and crucial variables for an up-scaling of this process required to be assessed after photocatalytic oxidation was shown to be a successful technique for destroying the contaminants found in the cosmetic effluent collected for this research. In addition to destroying water contaminants, the present technical use of UV/H_2_O_2,_ photo Fenton and photo Fenton-like oxidation processes aim to reduce the energy required for the complete degradation of the organic load. As a result, it is critical to investigate the economic parameter of photocatalytic oxidation processes, which is represented by energy consumption.

The energy needed to remove 70% of the initially COD of the wastewater used in this work utilizing the photochemical oxidation processes UV/H_2_O_2_, photo Fenton process, and photo Fenton-like process is generally compared in Table 3. Based on the amount of time required to remove 70% of the wastewater’s initial COD, the energy consumption of each photochemical process is calculated as kWh/kg. It should be noted that just the converted UV radiation which accounted for a small portion of the overall electrical energy was used to compute the specific energy consumed. 62 W of energy was produced. The following Eq. 9 can be used to estimate the specific energy consumption, E_s_ (kWh/Kg COD), required to remove 70% of wastewater’s COD^39^.

9 $$\:{E}_{s}=\frac{{P}_{UV}\:\times\:\:t}{\left[\varDelta\:COD\right]\times\:\:v}$$

where P_UV_ (kW) is the electrical power consumed by the UV lamp = 62 W and stirrer = 50 W, t (h) is the time at which 70% COD of wastewater was removed and equals 80, 20 and 40 min for the UV/H_2_O_2_, photo-Fenton and photo Fenton like processes respectively treatment, and V is the irradiated reaction volume (L), equals to 1 L, [ΔCOD] (kg) is the change in COD concentration of the wastewater and represents 70% COD removal.

The specific energy consumption for UV/H_2_O_2_, Photo Fenton and Photo Fenton Like were 30.39, 7.66 and 15.66 KWh/Kg respectively.

**Table 3:** Energy consumption required for COD removal per I L with cost.

Energy	Energy (KWh/KgCOD)			Cost for 1 L Treatment		
	UV/H_2_O_2_	Photo Fenton	photo Fenton like	UV/H_2_O_2_	Photo Fenton	photo Fenton like
UV	16.82	4.26	8.82	0.082418	0.020874	0.043218
Stirrer	13.57	3.40	6.84	0.066493	0.01666	0.033516
Total cost of Energy USD/L				0.148911	0.037534	0.076734

The energy consumption data in Table 3 are expressed in kWh per kg COD removed. To convert this into energy cost per liter (USD/L), the energy values were multiplied by the estimated COD removed per liter in (kg/L) and the electricity rate (USD/kWh).

Since each experiment treated 1 L of wastewater with an average COD removal efficiency of about 70%, the COD removed per liter was calculated based on the initial COD concentration and used in the conversion. This allowed comparison between energy and chemical costs, both expressed in USD/L.

The photo-Fenton and photo Fenton-like processes are more cost-effective than the UV/H_2_O_2_ process, with a four-fold reduction in energy for the photo Fenton system and a twofold reduction for the photo Fenton-like system. The photo-Fenton process is thought to be the most efficient and economical method to degrade the cosmetic wastewater that is being investigated in this research.

### Cost estimation

A comparison of the material costs associated with different advanced oxidation processes reveals substantial variations in economic feasibility is illustrated in Table 4. The UV/H₂O₂ process demonstrates the highest chemical cost per liter of treated wastewater, largely attributed to the high dosage of hydrogen peroxide required. Conversely, the Photo-Fenton and Photo-Fenton-like processes require lower reagent concentrations, significantly reducing the cost per liter. Specifically, the Photo-Fenton process incurs the lowest chemical expense (0.858 USD/L), slightly outperforming the Photo-Fenton-like process (0.862 USD/L).

**Table 4:** Ideal doses required for treating 1 L cosmetic wastewater with Cost.

Materials	Ideal Dose/L			Cost for 1 L Treatment		
	UV/H_2_O_2_	Photo Fenton	Photo Fenton like	UV/H_2_O_2_	Photo Fenton	Photo Fenton like
Hydrogen Peroxide 30%, L	5 mL/L	1mL/L	1mL/L	4.15	0.83	0.83
Ferrous Sulphate, Kg	----	0.75 g/L	----	----	0.02805	
Ferric Chloride, Kg	----	----	0.75 g/L	----	----	0.0325
Sulfuric Acid 98%, L	0.05 mL/L	0.05 mL/L	0.05 mL/L	0.00015	0.00015	0.00015
Sodium Hydroxide 48%, Kg	0.0213 g/L	0.0213 g/L	0.0213 g/L	0.0000713	0.0000713	0.0000713
Total cost of materials USD/L				4.1502213	0.8582713	0.8627213
The Overall Cost of Treatment USD/L				4.29913	0.89581	0.93946

The overall cost of treatment (USD/L) was calculated by combining two main components: energy cost and chemical cost, both normalized per liter of treated wastewater as shown in Eq. 10.

10 $$\:Overall\:Cost\:\left(\frac{USD}{L}\right)=Energy\:Cost\left(\frac{USD}{L}\right)+Chemical\:Cost\:\left(\frac{USD}{L}\right)$$

When both energy and material costs are considered, the overall treatment cost further highlights the advantage of the Photo-Fenton process. Despite similar chemical expenses between the iron-based processes, the markedly lower energy consumption of the Photo-Fenton system results in the most cost-effective solution for COD removal. Therefore, from both operational and economic perspectives, the Photo-Fenton method stands out as the optimal choice for enhancing cosmetic wastewater biodegradability.

When compared to conventional wastewater treatment methods, the cost of the Photo-Fenton process remains relatively higher. Typical biological or physicochemical treatments (e.g., coagulation-flocculation, activated sludge) incur operational costs ranging from 0.08 to 0.20 USD/L, depending on wastewater strength and infrastructure^40^.

The 0.89581 USD/L cost calculated in this study for Photo-Fenton reflects batch-scale operation and the complexity of real cosmetic wastewater. However, AOPs provide a distinct advantage in terms of removal of recalcitrant organic compounds and biodegradability enhancement, which conventional systems often fail to address. Thus, while the current cost may be higher, the process adds functional value and complementarity when integrated with biological post-treatment, especially for industries with complex effluents such as cosmetic industry.

## Development of an empirical regression model for COD removal efficiency with statistical analysis

An empirical regression model was developed using multiple linear regression analysis to describe the relationship between process parameters and COD removal efficiency. The model was constructed based on experimental data obtained under various operating conditions, including H₂O₂ dosage, Fe^+2^ dosage, pH, and irradiation time. The resulting equation represents the statistical correlation among these variables and their combined effect on the COD removal percentage. As this model below in Eq. (11) is derived directly from observed data.

11 $$\:\text{COD Removal}\:\text{\%}=-14.620+13.373\:\text{A}+154.609\:\text{B}-3.302\:\text{C}+1.084\:\text{D}-2.599\:{\text{A}}^{2}-90.402\:{\text{B}}^{2}$$

Where A is H_2_O_2_ dosage (mL/L), B is Fe^+2^ dosage (g/L), C is pH and D is irradiation time(min). The regression model demonstrated a strong predictive power, with a coefficient of determination R² of 0.851 and an adjusted R² of 0.837, indicating that approximately 85.1% of the variability in COD removal could be explained by the selected predictors. The ANOVA test shown in Table 5, confirmed the model’s statistical significance (F = 58.180, *p* < 0.001).

**Table 5:** ANOVA test results from photo Fenton Process.

	Sum of Squares	df	Mean Square	F	Significance
Regression	20608.264	6	3434.711	58.180	<.001^b^
Residual	3601.208	61	59.036		
Total	24209.472	67			

Table 6 revealed that all parameters except the intercept were statistically significant (*p* < 0.05), suggesting their notable influence on the COD removal efficiency. Specifically, the linear terms of H₂O₂, Fe^+2^, pH, and reaction time showed positive contributions, while the squared terms of H₂O₂ and Fe^+2^ had negative coefficients, indicating a nonlinear relationship, and suggesting optimal levels beyond which efficiency declines. Among these, Fe^+2^ concentration had the strongest positive effect (β = 2.721, *p* < 0.001), followed by time and H₂O₂.

**Table 6:** Statistical parameters for all Coefficients.

	Unstandardized Coefficients Beta	Standard Error	Standardized Coefficients Beta	t- State	*P*-value	Significance
Intercept	−14.620	7.848		−1.863	0.067	Not Significance
Variable 1	13.373	3.181	1.179	4.204	< 0.001	Significance
Variable 2	154.609	14.261	2.721	10.841	< 0.001	Significance
Variable 3	−3.302	1.449	− 0.128	−2.279	0.026	Significance
Variable 4	1.084	0.083	0.642	13.010	< 0.001	Significance
Variable 5	−2.599	0.523	−1.375	−4.970	< 0.001	Significance
Variable 6	−90.402	9.322	−2.440	−9.698	< 0.001	Significance

**Fig. 18:**
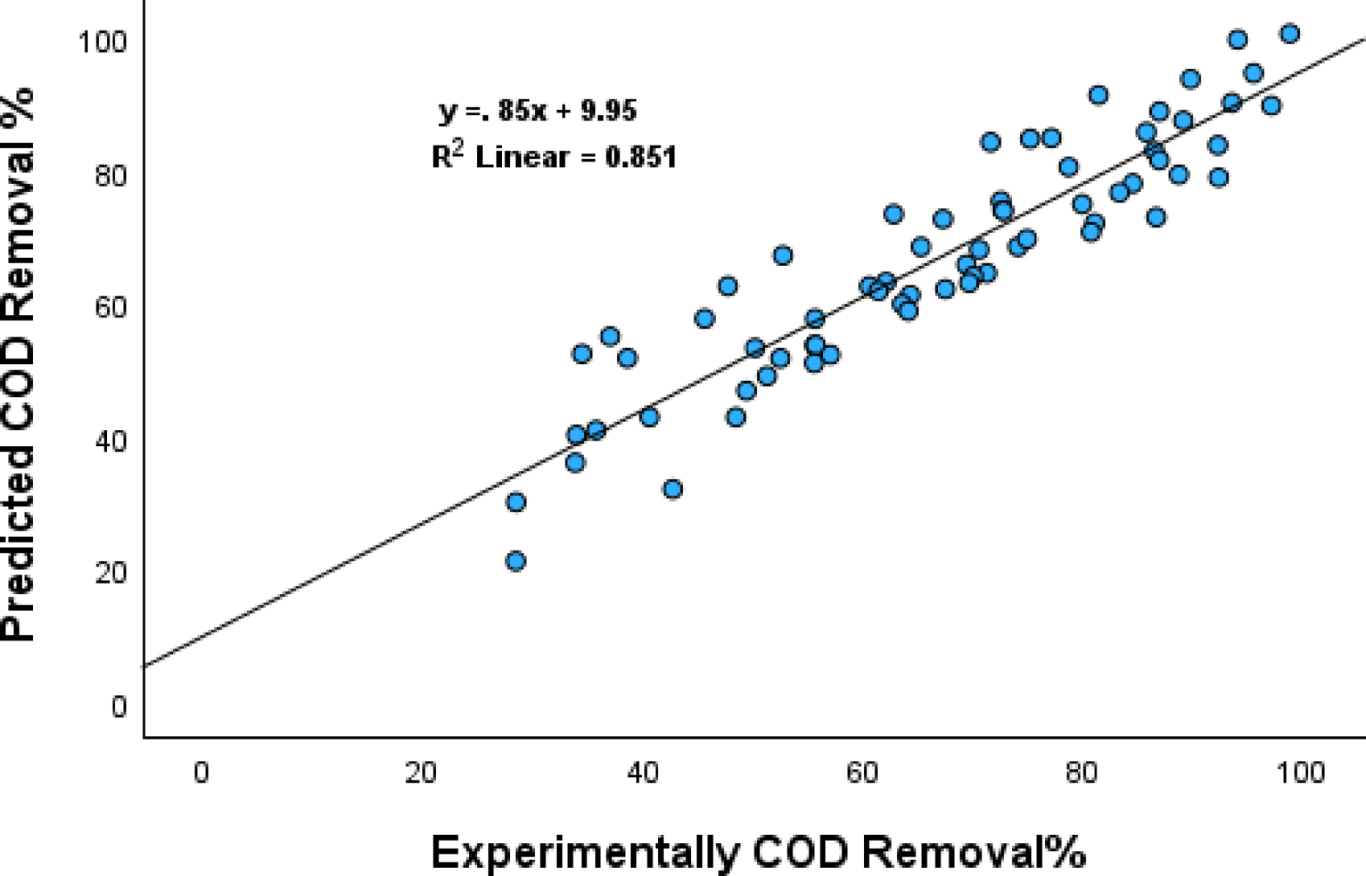
Relationship Between Experimentally COD Removal% and Predicted COD Removal%.

The scatter plot in Fig. 18 of experimental vs. predicted COD removal percentages illustrated a good agreement between the model and actual results, further validating the regression model’s accuracy (y = 0.85x + 9.95, R² = 0.851). These findings underscore the significance of optimizing reagent concentrations and irradiation time to maximize the performance of the Photo-Fenton process in wastewater treatment.

## Industrial applicability and future perspectives

This study was conducted using real industrial wastewater collected from a cosmetics factory located in Badr City, Cairo, Egypt. The experimental results demonstrated that the Photo-Fenton process, under optimized conditions (pH = 3, Fe^+2^ = 0.75 g/L, H₂O₂ = 1 mL/L), achieved the highest COD removal (95.5%) and biodegradability enhancement (BOD₅/COD =), making it the most efficient process among those tested.

The process was further validated through a multiple linear regression model with a high coefficient of determination (R² = 0.851), confirming the significant effect of operational variables on treatment efficiency. In addition, energy and cost assessments revealed that the Photo-Fenton process is not only effective but also economically viable, with the lowest energy consumption and material cost per liter treated.

The feasibility of scaling up such a process has been demonstrated in recent industrial applications. For example, Zárate Guzmán et al., 2020 reported the successful use of a continuous solar Photo Fenton system for piggery wastewater treatment, achieving over 98% organic removal. These findings support the scalability and industrial relevance of the current study and suggest that the proposed method can be adapted for larger-scale treatment in the cosmetics and related industries^41^.

Building on these outcomes, the Photo-Fenton process can serve as a cost-effective and compact pre-treatment step in decentralized industrial settings, particularly in regions with limited centralized infrastructure. The developed regression model offers predictive capabilities that enhance process adaptability across various wastewater types and conditions.

Although this study did not experimentally assess the reusability of iron-based catalysts, the regeneration and reuse of Fe^+2^/Fe^+3^ species have been reported in previous studies using techniques such as chemical reduction, precipitation followed by recovery, or immobilization on solid supports^42^. Including such strategies could improve the long-term sustainability and economic feasibility of the Photo-Fenton and Photo-Fenton-like processes.

Future research should explore catalyst recovery and regeneration protocols to minimize sludge production and reduce overall chemical consumption during repeated treatment cycles. Future research should also focus on the transition from batch to continuous flow system and the integration of AOPs with downstream biological treatments for enhanced overall sustainability and compliance with environmental discharge limits.

## Conclusion

This study demonstrated the effectiveness of four advanced oxidation processes (UV, UV/H₂O₂, Photo Fenton, and Photo Fenton like) for treating real wastewater from a cosmetics factory in Badr City, Egypt. Among the tested processes, the Photo-Fenton system showed the highest COD removal efficiency (95.5%) and significant improvement in biodegradability (BOD₅/COD = 0.80), highlighting its suitability as a robust pre-treatment method. The optimal operational conditions were identified as pH 3, 0.75 g/L of Fe^+2^, and 1 mL/L H₂O₂ for photo Fenton process.

Kinetic analysis indicated pseudo-first-order reaction behavior, with the photo Fenton process showing the highest rate constant. A multiple linear regression model (R² = 0.851) was successfully developed to predict COD removal based on operational conditions, supporting data-driven optimization. Additionally, economic and energy evaluations confirmed the Photo Fenton process as the most cost effective and sustainable option. These findings underscore the possibility of integrating AOPs with conventional wastewater treatment systems to meet stringent environmental regulations and reduce the hazardous impacts of industrial effluents.

This is considered of practical relevance for small and medium sized enterprises lacking centralized wastewater treatment systems, especially in developing regions. Scientifically, this work contributes to bridging the gap between lab-scale AOP research and full-scale industrial application, offering a validated empirical model and actionable treatment guidelines.

## Recommendations for future work

Future work should focus on scaling up the most promising AOPs, particularly the Photo-Fenton process, to pilot or continuous flow systems. Further studies are needed to assess the long-term performance and operational stability under real industrial conditions. Investigating the regeneration and reusability of iron-based catalysts, sludge management, and potential integration with biological or membrane-based post-treatment could enhance the overall sustainability and cost-effectiveness of the system. Additionally, applying statistical design methods such as response surface methodology (RSM) or Box–Behnken design could improve process optimization and interaction analysis.

In addition to the general recommendations, the following technical improvements are suggested for enhancing the efficiency, sustainability, and scalability of the Photo-Fenton process:

- Visible-light activation using broader spectrum sources or sunlight to improve energy efficiency and increase radical generation.
- Dual oxidant systems (e.g., combining H₂O₂ with peroxymonosulfate or peroxydisulfate) to boost degradation pathways and pollutant breakdown.
- Heterogeneous Photo Fenton systems using immobilized iron catalysts (e.g., Fe₃O₄ or Fe₂O₃ on silica or biochar) to reduce sludge and allow catalyst reuse.
- Gradual or controlled dosing of H₂O₂ during the reaction to minimize radical scavenging and increase treatment stability.

## Data Availability

The datasets used and analyzed during the current research are available from the corresponding author upon request.
